# Cyclophilin a signaling induces pericyte-associated blood-brain barrier disruption after subarachnoid hemorrhage

**DOI:** 10.1186/s12974-020-1699-6

**Published:** 2020-01-11

**Authors:** Pengyu Pan, Hengli Zhao, Xuan Zhang, Qiang Li, Jie Qu, Shilun Zuo, Fan Yang, Guobiao Liang, John H. Zhang, Xin Liu, Haiyang He, Hua Feng, Yujie Chen

**Affiliations:** 1 Department of Neurosurgery, Southwest Hospital, Army Medical University (Third Military Medical University), Chongqing, 400038 China; 2 Department of Neurosurgery, General Hospital of Northern Theater Command (Shenyang Military Command), Shenyang, 110016 China; 3 Department of Pathophysiology and High-Altitude Pathology, College of High-Altitude Military Medicine, Third Military Medical University, Chongqing, 400038 China; 4 Neuroscience Research Center, Loma Linda University, Loma Linda, California, 92350 USA; 5 Institute of Immunology, Army Medical University (Third Military Medical University), Chongqing, 400038 China; 6 State Key Laboratory of Trauma, Burn and Combined Injury, Third Military Medical University, Chongqing, 400038 China; 7 Chongqing Key Laboratory of Precision Neuromedicine and Neuroregenaration, Southwest Hospital, Third Medical University, Chongqing, 400038 China

**Keywords:** Subarachnoid Hemorrhage, Pericyte, Blood–brain barrier, Cyclophilin A, CD147

## Abstract

**Objective:**

The potential roles and mechanisms of pericytes in maintaining blood–brain barrier (BBB) integrity, which would be helpful for the development of therapeutic strategies for subarachnoid hemorrhage (SAH), remain unclear. We sought to provide evidence on the potential role of pericytes in BBB disruption and possible involvement and mechanism of CypA signaling in both cultured pericytes and SAH models.

**Methods:**

Three hundred fifty-three adult male C57B6J mice weighing 22 to 30 g, 29 CypA^−/−^ mice, 30 CypA^+/+^ (flox/flox) mice, and 30 male neonatal C57B6J mice were used to investigate the time course of CypA expression in pericytes after SAH, the intrinsic function and mechanism of CypA in pericytes, and whether the known receptor CD147 mediates these effects.

**Results:**

Our data demonstrated both intracellular CypA and CypA secretion increased after SAH and could activate CD147 receptor and downstream NF-κB pathway to induce MMP9 expression and proteolytic functions for degradation of endothelium tight junction proteins and basal membranes. CypA served as autocrine or paracrine ligand for its receptor, CD147. Although CypA could be endocytosed by pericytes, specific endocytosis inhibitor chlorpromazine did not have any effect on MMP9 activation. However, specific knockdown of CD147 could reverse the harmful effects of CypA expression in pericytes on the BBB integrity after SAH.

**Conclusions:**

This study demonstrated for the first time that CypA mediated the harmful effects of pericytes on BBB disruption after SAH, which potentially mediated by CD147/NF-κB/MMP9 signal, and junction protein degradation in the brain. By targeting CypA and pericytes, this study may provide new insights on the management of SAH patients.

## Introduction

Despite years of research, early brain injury, which is the main contributor to the mortality and poor prognosis of patients after subarachnoid hemorrhage (SAH), remains a worrisome complication of ruptured intracranial aneurysms [1]. The pathophysiology of SAH and other stroke events involves changes in the cerebral vascular neural network, including the arterial and venous systems as well as neuronal cells, other support cells, and cellular matrices [2]. Within this vascular neural network, blood–brain barrier damage contributes to vasogenic brain edema, which is one of the most common pathophysiological changes after SAH and has become a focus of interest in the development of novel therapeutic strategies for SAH patients. However, the exact mechanism underlying blood–brain barrier disruption and remodeling has not been clarified.

Pericytes are partially sandwiched between endothelial cells and astrocyte end feet and represent important constituents of the neurovascular unit, and they have traditionally been considered essential for normal blood–brain barrier function [3]. A previous study indicated that pericytes bidirectionally control microvessel diameters and regulate cerebral blood flow [4]. Our previous study further demonstrated that pericyte α-SMA phenotype transformation caused acute microvessel/pearl-like constriction and neurological deficits in the setting of SAH [5, 6]. However, a recent study suggested that pericytes might have a negative influence on neurovascular integrity and cause neuronal degeneration in apolipoprotein E-deficient mice [7]. More direct evidence was found using a two-photon microscope, which showed that pericytes contribute to rapid and localized proteolytic degradation around pericyte somata during ischemia [8].

Therefore, as a multifunctional cell type, the pathophysiological changes in pericytes after SAH should be further investigated [9]. Cyclophilin A (CypA), which is encoded by the peptidylprolyl isomerase A gene, is a small molecule protein located in the cytoplasm and the extracellular space, and it has been widely used as a target of immunosuppressant after organ transplantation for anti-rejection purposes as well as in the treatment of autoimmune diseases [10]. Our previous work also indicate that CypA-specific inhibitor, Cyclosporine A, could alleviate MMP9-associated blood–brain barrier disruption [11]. Recent studies have reported that CypA in pericytes is actually the core of blood–brain barrier integrity in neurodegenerative diseases [7]. However, the potential roles of pericytes in maintaining the integrity of the blood–brain barrier following SAH and the exact mechanisms of this process, which would be helpful for the development of therapeutic strategies, remain unclear. In the present study, we sought to provide additional evidence on the potential role of pericytes in blood–brain barrier disruption and the possible involvement and mechanism of CypA signaling in both cultured primary pericytes and SAH models.

## Materials and methods

### Animals

Three hundred fifty-three adult male C57B6J mice weighing 22 to 30 g, 29 CypA knockout (CypA^−/−^) mice, 30 CypA^+/+^ (flox/flox) mice, and 30 male neonatal C57B6J mice were provided by the Experimental Animal Center of the Third Military Medical University (Chongqing, China) for the present study. CypA^−/−^ mice were purchased form The Jackson Laboratory (ME, USA) and backcrossing at least ten generations into C57B6J background. All experimental procedures were approved by the Ethics Committee of Southwest Hospital and performed in accordance with the guidelines in the National Institutes of Health Guide for the Care and Use of Laboratory Animals and followed the ARRIVE guidelines.

### Experimental design

The present study contained four experiments (Additional file 1: Figure S1), which were designed as follows.

### Experiment I

To determine the time course of CypA expression in pericytes after SAH, 44 mice were randomly assigned into seven groups: Sham (*n* = 7), SAH 3 h (*n* = 6), SAH 6 h (*n* = 6), SAH 12 h (*n* = 6), SAH 24 h (*n* = 7), SAH 48 h (*n* = 6), and SAH 72 h (*n* = 6). Western blots were used to detect the CypA protein expression in microvessels isolated from the ipsilateral/left hemisphere in each group. Immunohistochemical staining of CypA, PDGFRβ/CD13, and Lectin was performed 24 h after SAH induction to confirm the spatial distribution of CypA in the pericytes (*n* = 2). None of the sham-operated mice died, and eight mice died within 72 h and after SAH caused by severe hemorrhagic volume.

### Experiment II

To define the intrinsic function of CypA in the pericytes, 30 CypA^+/+^ (flox/flox) adult C57B6J mice and 29 CypA^−/−^ mice were randomly assigned into four groups: flox/flox + Sham (*n* = 13), flox/flox + SAH (*n* = 13), KO + Sham (*n* = 13), and KO + SAH (*n* = 13) groups. Then, modified Garcia tests and beam balance tests were performed 24 h after SAH induction to evaluate the neurological deficits in each group (*n* = 6). In addition, an Evans blue extravasation assessment and fluorescence imaging of Evans blue and Cadaverine extravasation (*n* = 6) were performed 24 h after SAH induction to detect the blood–brain barrier disruptions. Immunohistochemical staining was also performed to detect the spatial expression of collagen IV and Lectin in the ipsilateral/left hemisphere 24 h after SAH induction (*n* = 2). None of the sham-operated mice died, and three CypA^+/+^ (flox/flox) mice and two CypA^−/−^ mice died after SAH caused by severe hemorrhagic volume.

Furthermore, 155 wild-type adult C57B6J mice were randomly divided into the following groups: Sham (*n* = 31); SAH + vehicle (2 μl of sterile saline; *n* = 31), SAH + CypA (200 ng in 2 μl of sterile saline; *n* = 31); SAH + scrambled small interfering RNA (SAH + Scr siRNA; 500 pmol in a 2-μl mixture of 1:1 DEPC-treated water and liposome; *n* = 31); and SAH + CypA small interfering RNA (SAH + CypA siRNA; RiboBio, Guangzhou, China; 500 pmol in a 2-μl mixture of 1:1 DEPC-treated water and liposome; *n* = 31). Scrambled siRNA or CypA siRNA was intracerebroventricularly injected at 48 h before SAH. Modified Garcia tests (*n* = 6), beam balance tests (*n* = 6), brain water content assessment (*n* = 6), and Evans blue extravasation assessment (*n* = 6) were performed 24 h after SAH induction. Immunohistochemical staining was also performed to detect the spatial expression of collagen IV and lectin in the ipsilateral/left hemisphere 24 h after SAH induction (*n* = 1). Western blots were performed to detect the P-p65 and MMP9 protein expression in microvessels isolated from the ipsilateral/left hemisphere of each group (*n* = 6); and the ZO-1, collagen IV, Occludin, and claudin 5 expression in the total cortex protein (*n* = 6). In addition, gelatin zymography (*n* = 6) was used to detect the proteolytic capacity of MMP9. None of the sham-operated mice died, and 63 mice died within 72 h and after SAH caused by severe hemorrhagic volume.

### Experiment III

To investigate the secretion of CypA from the pericytes after SAH, 30 neonatal mice were used for the primary pericyte culture. To determine the time course of the intracellular and extracellular CypA expression in the pericytes after oxyhemoglobin (Hb) stimulation, cultured primary pericytes were randomly assigned into seven groups: Vehicle (*n* = 6), Hb 3 h (*n* = 6), Hb 6 h (*n* = 6), Hb 12 h (*n* = 6), Hb 24 h (*n* = 6), Hb 48 h (*n* = 6), and Hb 72 h (*n* = 6). Western blots were performed to detect the protein expression of intracellular CypA in the pericytes of each group, and the results were confirmed via immunohistochemical staining at 24 h after Hb stimulation. The supernatants of the culture media were collected to measure the secreted CypA via ELISA.

To determine whether endocytosis of CypA occurs in the pericytes and to screen for the inhibitors of CypA endocytosis, exogenous CypA was conjugated with FITC to investigate the endocytosis of CypA in the cultured pericytes, and laser confocal scanning of CypA-FITC was performed in the cultured primary pericytes at 10 min, 30 min, 1 h, 2 h, and 3 h after adding CypA-FITC (1 μg/ml, Novoprotein, Shanghai, China) to the culture media. In addition, the cultured primary pericytes were randomly assigned to seven groups: DMSO (*n* = 6); DMSO + CypA-FITC (1 μg/ml) (*n* = 6); Amiloride hydrochloride (100 μM, Sigma-Aldrich, Shanghai, China) + CypA-FITC (1 μg/ml) (*n* = 6); Dynasore (80 μM, Sigma-Aldrich, Shanghai, China) + CypA-FITC (1 μg/ml) (*n* = 6); Genistein (200 μM, Sigma-Aldrich, Shanghai, China) + CypA-FITC (1 μg/ml) (*n* = 6); Chloroquine diphosphate (200 μM, Sigma-Aldrich, Shanghai, China) + CypA-FITC (1 μg/ml) (*n* = 6); and Chlorpromazine hydrochloride (30 μM, Sigma-Aldrich, Shanghai, China) + CypA-FITC (1 μg/ml) (*n* = 6). Flow cytometry was used to detect the FITC-positive cells percentage in each group.

To determine whether secretion inhibitor Exo1 and Exo2 reducing CypA secretion from pericytes, cultured primary pericytes were randomly assigned to five groups: DMSO (*n* = 6); Hb (*n* = 6); Hb + vehicle (*n* = 6); Hb + Exo1 (100 μM, Sigma-Aldrich, Shanghai, China) (*n* = 6); and Hb + Exo2 (50 μM, Sigma-Aldrich, Shanghai, China) (*n* = 6). Secreted CypA and MMP9 in the medium were detected by ELISA.

### Experiment IV

To determine whether the known receptor CD147 mediates the effects of CypA, the following experiments were performed. First, to determine the time course of CD147 expression in pericytes after SAH, 44 mice were randomly assigned into seven groups: Sham (*n* = 7), SAH 3 h (*n* = 6), SAH 6 h (*n* = 6), SAH 12 h (*n* = 6), SAH 24 h (*n* = 7), SAH 48 h (*n* = 6), and SAH 72 h (*n* = 6). Western blots were performed to detect the CD147 protein expression in microvessels isolated from the ipsilateral/left hemisphere of each group. Immunohistochemical staining of CD147, PDGFRβ/CD13, and Lectin was performed 24 h after SAH induction to confirm the spatial distribution of CD147 in the pericytes (*n* = 1). In addition, cultured primary pericytes were randomly assigned into seven groups: Vehicle (*n* = 6), Hb 3 h (*n* = 6), Hb 6 h (*n* = 6), Hb 12 h (*n* = 6), Hb 24 h (*n* = 6), Hb 48 h (*n* = 6), and Hb 72 h (*n* = 6). Western blots were performed to detect the protein expression level of intracellular CD147 in the pericytes of each group, and the results were confirmed via immunohistochemical staining 24 h after Hb stimulation. All mice and cultured pericytes specimen in experiment III were from experiment I.

Second, to determine whether CD147 play a role in endocytosis of CypA by pericyte, cultured primary pericytes were randomly assigned into four groups: DMSO (*n* = 6); DMSO + CypA-FITC (1 μg/ml) (*n* = 6); CypA-FITC (1 μg/ml) + scrambled siRNA; and CypA-FITC (1 μg/ml) + CD147 siRNA (RiboBio, Guangzhou, China). Flow cytometry was used to detect the FITC-positive cells percentage in each group. Then, to detect MMP9 secretion, cultured pericytes were randomly assigned into nine groups: DMSO; Hb (10 μM) + DMSO; Hb + CypA (100 ng/ml); Hb + scrambled siRNA; Hb + CypA siRNA; Hb + CypA + scrambled siRNA; Hb + CypA + CD147 siRNA; Hb + CypA + DMSO; and Hb + CypA + chlorpromazine (30 μM). An ELISA was performed to measure the level of MMP9 in the supernatant of the culture media, and gelatin zymography was used to detect its proteolytic capacity.

Third, to validate the role of CD147 after SAH in vivo, 125 mice were randomly assigned into five groups: Sham (*n* = 25); SAH + vehicle (2 μl of sterile saline; *n* = 25); SAH + CypA (200 ng in 2 μL of sterile saline; *n* = 25); SAH + CypA (200 ng in 2 μl of sterile saline) + scrambled siRNA (500 pmol in a 2-μl mixture of 1:1 DEPC-treated water and liposome; *n* = 25); and SAH + CypA (200 ng in 2-μl sterile saline) + CD147 siRNA (500 pmol in a 2-μl mixture of 1:1 DEPC-treated water and liposome; *n* = 25). Scrambled siRNA or CypA siRNA was intracerebroventricularly injected 48 h before SAH. Modified Garcia tests (*n* = 6), beam balance tests (*n* = 6), brain water content assessments (*n* = 6), and Evans blue extravasation assessments (*n* = 6) were performed 24 h after SAH induction. Immunohistochemical staining was also performed to detect the spatial expression of collagen IV and Lectin in the ipsilateral/left hemisphere 24 h after SAH induction (*n* = 1). Western blots (*n* = 6) were performed to detect the P-p65 and MMP9 protein expression in microvessels isolated from the ipsilateral/left hemisphere of each group; and ZO-1, collagen IV, Occludin, and claudin 5 expression was detected in the total cortex protein. In addition, gelatin zymography (*n* = 6) was used to detect the proteolytic capacity of MMP9. None of the sham-operated mice died, and 28 mice died within 72 h and after SAH caused by severe hemorrhagic volume.

### SAH model

The SAH mouse model was generated by endovascular perforation as previously described [12]. Briefly, under sodium pentobarbital anesthesia (40 mg/kg, intraperitoneally injected), the external carotid artery was identified and distally transected into a 2-mm stump. A 5-0 sharpened monofilament nylon suture was advanced into the internal carotid artery through the external carotid artery until resistance was felt, and then it was pushed 2 mm further to penetrate the bifurcation of the anterior and middle cerebral artery. The suture was then withdrawn, and the internal carotid artery was reperfused to produce the SAH. Sham-operated mice underwent the same procedure, although the suture was withdrawn without perforating the cerebral artery after feeling resistance.

### Intracerebroventricular injection

The intracerebroventricular injection procedure was performed as previously described [13]. A small burr hole was drilled 1.0 mm lateral to the bregma on the skull. The needle of a 10-μl Hamilton syringe (Microliter 701; Hamilton Company, Reno, NV, USA) was stereotactically inserted into the left lateral ventricle through the burr hole 3.0 mm below the horizontal plane of the bregma. A 2 μl volume of CypA (Novoprotein, Shanghai, China) in sterile saline was infused at a rate of 0.2 μl/min 1 h after SAH induction, whereas 500 pmol/2 μl of CypA, CD147, or scrambled siRNA was infused at the same rate 48 h before SAH modeling. The syringe was left in situ for an additional 10 min before slowly removing it from the animal.

CypA siRNA and CAD147 siRNA are pools of three different siRNA duplexes in order to improve the knockdown efficiency.

Targeting sequences of CypA siRNA are provided in 5′→3′ orientation as follows:

(I) TGACTTTACACGCCATAAT
(II) CCATCTACGGAGAGAAATT
(III) GCATCTTGTCCATGGCAAA

Targeting sequences of CD147 siRNA are provided in 5′→3′ orientation as follows:

(I) GGATCAAGGTCGGAAAGAA
(II) GCAAGTCCGATGCATCCTA
(III) TCAGCAACCTTGACGTAAA

### Neurological outcome assessment

Neurological deficits were evaluated 24 and 72 h after SAH induction using the modified Garcia scale, which is an 18-point score system, and the beam balance test, which is a 5-point score system, in the outcome study [13]. The modified Garcia assessment consisted of six tests covering spontaneous activity, spontaneous movement of four limbs, forepaw outstretching, climbing, body proprioception, and response to whisker stimulation (3–18 points). For the beam balance test, the mice were placed on a beam and their walking distance within 1 min (0–5 points) was observed. The mean of the neurological score was evaluated by two blinded observers for grading.

### SAH grade assessment

An 18-point SAH severity grading system was used as previously described [14]. The basal cistern was divided into six segments that could be scored from 0 to 3 according to the amount of subarachnoid blood clotting. The total score was calculated by adding the scores from six segments (0–18 points). Animals that received a score < 8 were excluded from the study.

### Brain water content

The brains were quickly separated into left and right cerebral hemispheres, cerebellum, and brain stem and then weighed (wet weight). Then, the brain samples were dried in an oven at 55 °C for 48 h and weighed again (dry weight). The percentage water content was calculated as follows: ([wet weight − dry weight]/wet weight) × 100%.

### Extravasation and fluorescence of Evans blue and Cadaverine-Alexa Flour 555

Evans blue extravasation was performed as previously described [6]. At 24 h post-operation, Evans blue dye (2%, 5 ml/kg; Sigma–Aldrich, St. Louis, MO, USA) or Cadaverine-Alexa Flour 555 (500 μg/kg; Thermo Fisher Scientific, Waltham, MA, USA) was injected and administered > 2 min into the left femoral vein and allowed to circulate for 60 min. Under anesthesia, the mice were euthanized by an intracardial perfusion of phosphate-buffered saline (PBS). Then fluorescent efficiency of the Evans blue and Cadaverine extravasation was measured by a living image system, the IVIS Spectrum CT (PerkinElmer, Waltham, MA, USA) equipped with fluorescent filter sets (Evans blue, excitation/emission, 470/680 nm; Cadaverine-Alexa Flour 555, excitation/emission, 550/570 nm) and average efficiency of Cadaverine-Alexa Flour 555 was calculated by IVIS living image 4.5 software (PerkinElmer, Waltham, MA, USA). Subsequently, the brains were removed and divided into left and right cerebral hemispheres, and then the brain samples were weighed, homogenized in saline, and centrifuged at 15,000 g for 30 min. Next, an equal volume of trichloroacetic acid was added to the resultant supernatant. The samples were then incubated overnight at 4 °C and centrifuged at 15,000 g for 30 min. The resultant supernatant was then spectrophotometrically quantified for the extravasated Evans blue dye at 610 nm.

### Isolation of microvessels

Microvessels were isolated using dextran gradient centrifugation followed by sequential cell-strainer filtrations as previously described [15]. First, the mouse brain was isolated, and the meninges were removed in ice-cold PBS containing 2% fetal bovine serum (FBS). The cortex was collected, and all macroscopically visible white matter was removed. The cortex was then homogenized in FBS with a glass dounce homogenizer. Added Dextran (70 kDa, Sigma-Aldrich, Shanghai, China) into the samples to yield a final concentration of 16%, and the samples were thoroughly mixed sequentially. The samples were then centrifuged at 6000 g for 15 min. The microvessel pellet located at the bottom of the tubes was collected and filtered through 100 m and 45 m cell strainers (BD, Franklin Lakes, NJ, USA). The microvessels that remained on top of the 45 m cell strainer were collected in PBS for further analysis.

### Western blot

Western blot assays were performed as previously described [12, 16]. The protein extracted from the left hemisphere (perforation side) was used for the Western blot analysis. Equal amounts of total protein (30 μg) were loaded in each lane of SDS-PAGE gels. After gel electrophoresis, the protein was transferred onto a nitrocellulose membrane, which was then blocked via blocking buffer for 2 h at room temperature. The following primary antibodies were diluted to incubate with the membrane under gentle agitation at 4 °C overnight: anti-CypA (1:500; Abcam, Cambridge, UK), anti-CD147 (1:500; Abcam, Cambridge, UK), collagen IV (1:500; Abcam, Cambridge, UK), anti-ZO-1 (1:500; Invitrogen, Waltham, MA, USA), anti-Occludin (1:500; Invitrogen, Waltham, MA, USA), claudin 5 (1:500; Thermo Fisher Scientific, Waltham, MA, USA), P-p65 (1:500; Abcam, Cambridge, UK), p65 (1:500; Abcam, Cambridge, UK), and MMP9 (1:500; Abcam, Cambridge, UK). GAPDH and β-actin were used as internal loading controls and detected by anti-GAPDH and anti-β-actin primary antibodies (1:2000; Santa Cruz Biotechnology, Santa Cruz, CA, USA). The appropriate secondary antibodies were incubated with the nitrocellulose membrane for 2 h at room temperature. Chemiluminescent detection was performed to identify the immune bands (ECL Plus; Amersham Bioscience, Arlington Heights, IL, USA). Data were analyzed by densitometry using Quantity One 4.6.2 (Bio-Rad Laboratories, Berkeley, CA, USA).

### Immunofluorescence staining

Immunofluorescence staining was performed on fixed frozen brain sections as previously described [6, 17]. At 24 h after SAH induction, the mice were deeply anesthetized and transcardially perfused with PBS and 4% PFA. The brains were rapidly isolated and postfixed in 4% PFA for 24 h and then soaked in 40% sucrose for 1 day. Coronal brain sections (10 μm) were obtained using a cryostat (Leica CM3050S-3-1-1, Bannockburn, IL, USA) and permeabilized with 0.3% Triton X-100 in PBS for 30 min. Sections were blocked with 5% donkey serum for 1 h and incubated at 4 °C overnight with anti-collagen IV (Abcam, Cambridge, UK) and Lectin antibodies (Vector Laboratories, Burlingame, CA) and then incubated with fluorescein isothiocyanate-conjugated secondary antibodies (Jackson Immunoresearch, West Grove, PA) and Dylight 649-conjugated streptavidin (Vector Laboratories, Burlingame, CA) for 4 h at room temperature.

Brain capillaries were isolated as described above and then cytospun (500 g) for 5 min onto Superfrost Plus precleaned glass microscopy slides. The microvessel fragments were then fixed using ICC Fixation Buffer (BD, Franklin Lakes, NJ, USA) for 15 min at room temperature. The microvessels were then rinsed with PBS and blocked in PBS containing 0.4% Triton X-100 and 5% Swine Serum (Vector Laboratories, Burlingame, CA, USA) for 1 h at room temperature followed by incubation with the following antibodies: anti-CypA (Abcam, Cambridge, UK), anti-PDGFRβ (1:100, Abcam, Cambridge, United Kingdom), anti-CD13 (Novus Biologicals, Littleton, CO, USA), and anti-lectin (Vector Laboratories, Burlingame, CA, USA). The microvessels were subsequently incubated with fluorescein isothiocyanate-conjugated and Cy3-conjugated secondary antibodies (Jackson Immunoresearch, West Grove, PA, USA) as well as Dylight 649-conjugated streptavidin (Vector Laboratories, Burlingame, CA, USA) for 4 h at room temperature. The colocalization of CypA, the pericyte marker CD13 and Lectin was examined by a confocal laser scanning microscope (Zeiss, Oberkochen, Germany).

### Brain pericyte cultures and RNAi

Pericyte culture was performed as previously described [6]. The brains were harvested from 30 mice (P1–P3) and digested in an enzymatic solution that contained 30 U/ml papain and 40 μg/ml DNase I in HEPES balanced salt solution. Then, digestion was terminated in PBS containing 1.7 times the volume of 22% bovine serum albumin and centrifuged at 6000 g for 10 min. Cells in the lower layer were resuspended in endothelial cell growth medium consisting of Hams F12 supplemented with 10% FBS, heparin, ascorbic acid, l-glutamine, penicillin/streptomycin, and endothelial cell growth supplement (ScienCell Research Laboratories, San Diego, CA, USA). Then, these cells were plated in a six-well plate coated with collagen I (0.02%, Sigma-Aldrich, St. Louis, MO, USA) for 2 h at 37 °C. The first passage was performed on the 7th–9th day. Subsequently, the cells were cultured in pericyte medium (ScienCell Research Laboratories, San Diego, CA, USA) containing 2% FBS and could be used after the third passage. CypA siRNA, CD147 siRNA, or scrambled siRNA at a final concentration 100 nmol/ml; Lipofectamine 2000 Transfection Reagent (Thermo Fisher Scientific, Waltham, MA, USA) at a final concentration 0.4 vol.%; and Opti-MEM (Thermo Fisher Scientific, Waltham, MA, USA) at a final concentration 10 vol.% were mixed with serum-free medium, according to the manufacturer’s instructions. After incubation for 4 h, the siRNA-containing medium was replaced with the standard pericyte culture medium, WB, zymography, Flow cytometry, ELISA was performed respectively at 24 h post-treatment. For identification via immunocytochemical analysis, the cells were treated under the same conditions as described above but then passaged onto collagen-coated slides and grown for 3 days unless otherwise indicated. Cultures were confirmed to be morphologically consistent with pericyte cultures, and they were also PDGFRβ-positive, SMA-positive, CD13-positive, GFAP-negative, AQP4-negative, TUJ1-negative, NeuN-negative, CD31-negative, Iba1-negative, and Olig-2-negative as previously reported [6, 9].

### Gelatinase zymography

Gelatinase zymography was performed as previously described with modifications [18]. Briefly, isolated microvessels were homogenized in lysis buffer, and the pericytes conditioning media (1.5 ml) was concentrated to 20–30 μl using microcon spin columns (Millipore, Bedford, MA). The samples were separated on an 8% polyacrylamide SDS gel containing 1 mg/ml gelatin. The gel was then soaked in zymogram renaturing buffer (Novex, Shanghai, China) for 30 min at room temperature and incubated for 16 h at 37 °C in zymogram developing buffer (Novex, Shanghai, China). To visualize the MMPs, the gel was stained with SimplyBlue Safe Stain (Invitrogen, Shanghai, China) for 30 min. The gel was imaged using a CCD camera and analyzed using ImageJ software (NIH, Bethesda, MD, USA).

### Flow cytometry

The cells were incubated with the blank control, vehicle (DMSO), Amiloride (100 μM), Dynasore (80 μM), Genistein (200 μM), Chloroquine (200 μM), and Chlorpromazine (30 μM) for 1 h, and scrambled siRNA or CD147 siRNA was transfected for 48 h. The cells were then washed and incubated with CypA-FITC (1 μg/ml, Novoprotein, Shanghai, China) for 1 h except for the blank group. The cells were harvested in aliquots of 1 × 10^6^ cells per 100 μl. The positive cell count and FITC-positive cells percentage were determined via flow cytometry (BD, Franklin Lakes, NJ, USA). The data were processed using FlowJo 7.6.1 (BD, Franklin Lakes, NJ, USA).

### ELISA

The levels of CypA and MMP9 in the supernatant of the cultured pericyte samples were measured by ELISA according to the manufacturer’s protocol for CypA (CusaBio, Wuhan, China) and MMP9 (R&D system, Minneapolis, MN, USA) detection. The cells were treated with either a vehicle (sterile saline), Hb (10 μM), Hb + vehicle, Hb + CypA, Hb + scrambled siRNA, Hb + CypA siRNA, Hb + CypA + scrambled siRNA, Hb + CypA + CD147 siRNA, Hb + CypA + vehicle (DMSO), or Hb + CypA + CPZ (30 Μm). For the measurement of MMP9, the cells were pretreated for 48 h with scrambled siRNA, CypA siRNA, or CD147 siRNA, and the other inhibitors were added 24 h before collecting the cell supernatants. A total of 1 × 10^6^ cells were dispensed in triplicate onto 12-well plates and cultured for different times according to the manufacturer’s recommendations.

### Statistical analysis

Data are shown as the mean ± SD. Chi-square tests were used for the behavior score analyses. A one-way analysis of variance (ANOVA) followed by Tukey’s multiple comparisons test was used to compare the different groups. SPSS 18 was used to analyze the data, and *P* < 0.05 was considered significant.

## Results

### CypA expression in pericytes after SAH

**Fig. 1:**
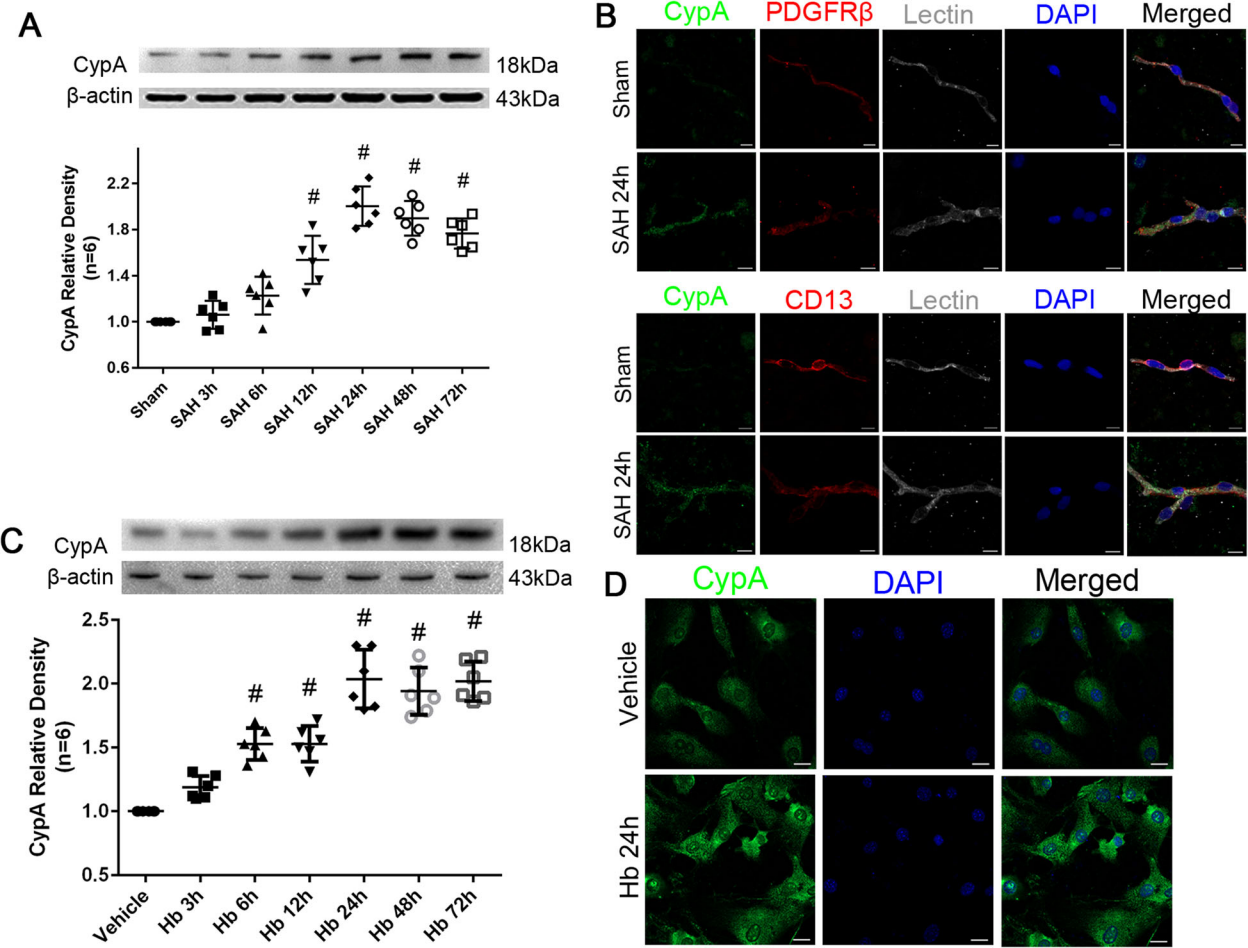
Time course of endogenous CypA in pericyte after subarachnoid hemorrhage. **a** Representative western blot bands and quantitative analysis of CypA from ipsilateral hemisphere after SAH. **b** Representative immunohistochemistry staining slices of CypA (Green), PDGFRβ/CD13 (Red), and Lectin (White) at 24 h after SAH. **c** Representative western blot bands and quantitative analysis of CypA expressions in cultured pericytes after Hb stimulation. **d** Representative immunohistochemistry staining of CypA (Green) in cultured pericytes at 24 h after Hb stimulation. Relative densities of each protein have been normalized against the sham group. *n* = 6 in panel **a**, *n* = 6 in panel **c**; #: vs. sham *P* < 0.05

To investigate the expression of CypA in the pericytes after SAH induction, we detected the protein levels of CypA in the microvessels of the brain specimen from the mice after SAH induction and in the cultured pericytes after Hb stimulation. The Western blot analysis illustrated a significant elevation of CypA in the ipsilateral/left hemisphere 12 to 72 h after SAH (Fig. 1a). Furthermore, immunohistochemical staining at 24 h (the peak of CypA expression) after SAH showed that CypA expression was colocalized with the pericyte markers, Lectin and PDGFRβ/CD13, in the left/ipsilateral cortex (Fig. 1b). The Western blot analysis of the cultured pericytes also demonstrated a significant elevation of intracellular CypA after Hb stimulation at all time points up to 72 h except at 3 h (Fig. 1c), and confirmed by immunohistochemical staining at 24 h after Hb stimulation (Fig. 1d).

### Neurological deficit changes and blood–brain barrier disruption after SAH in CypA^−/−^ mice

**Fig. 2:**
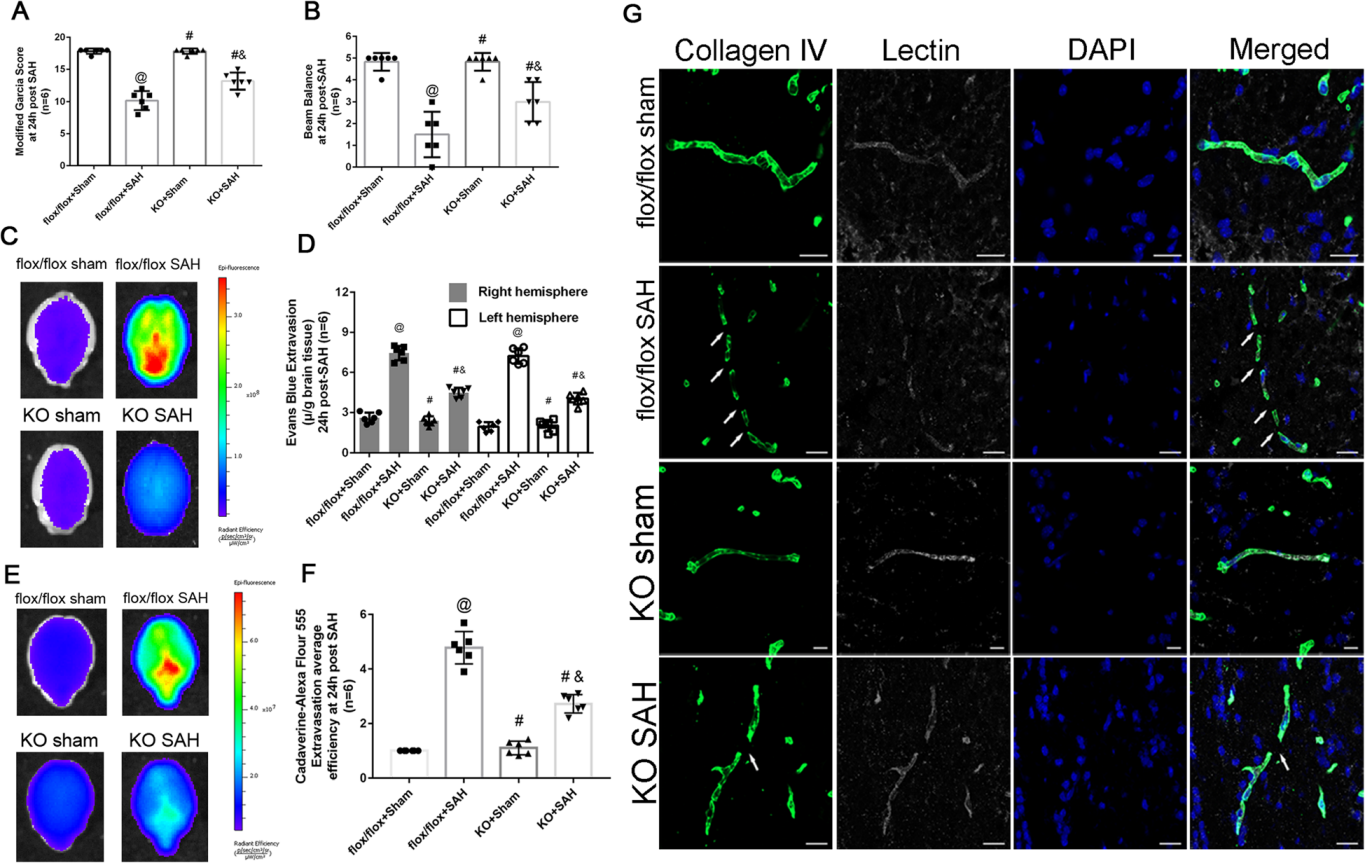
Neurological outcomes and blood brain barrier permeability after subarachnoid hemorrhage in CypA^−/−^ mice. **a** Modified Garcia test results of each group at 24 h after SAH. **b** Beam balance test results of each group at 24 h after SAH. **c** Evans blue fluorescence evaluation. **d** extravasation at 24 h after SAH. **e** Cadaverine-Alexa Flour 555 fluorescence evaluation. **f** Fluorescent efficiency at 24 h after SAH. **g** Representative immunohistochemistry staining slices of Collagen IV (Green) and Lectin (White) at 24 h after SAH. *n* = 6 for each group @: vs. flox/flox + sham *P* < 0.05, #: vs. flox/flox + SAH *P* < 0.05, &: vs. KO + sham, *P* < 0.05. Arrow indicates the breakdown of continuous endothelia cell layer

To confirm the pivotal role of CypA, we utilized CypA knockout (CypA^−/−^) mice in the present study. There was no statistical difference of SAH grade between wildtype and CypA^−/−^ mice in sham and SAH groups (Fig. 2a), which indicated the SAH models were consistent and comparable. The SAH mouse model exhibited significant neurological deficits at 24 h after SAH (Fig. 2a, b). However, compared with the flox/flox SAH mice, the CypA^−/−^ mice exhibited a better performance in the modified Garcia test (Fig. 2a) and the beam balance test (Fig. 2b). Further investigation indicated that the Evans blue and cadaverine extravasation in the CypA^−/−^ mice was less than that in the CypA^+/+^ (flox/flox) mice 24 h after SAH (Fig. 2c, e, f), which was verified by the fluorescence imaging of the Evans blue extravasation at the same timepoint after SAH (Fig. 2d). Immunohistochemical staining showed that the continuous endothelial cell layer (Lectin) and basement membrane (Collagen IV) were disrupted in wildtype SAH mice at 24 h after SAH, which was alleviated in CypA^−/−^ SAH mice (Fig. 2e).

### Effects of exogenous recombinant CypA and specific inhibition of CypA expression after SAH

**Fig. 3:**
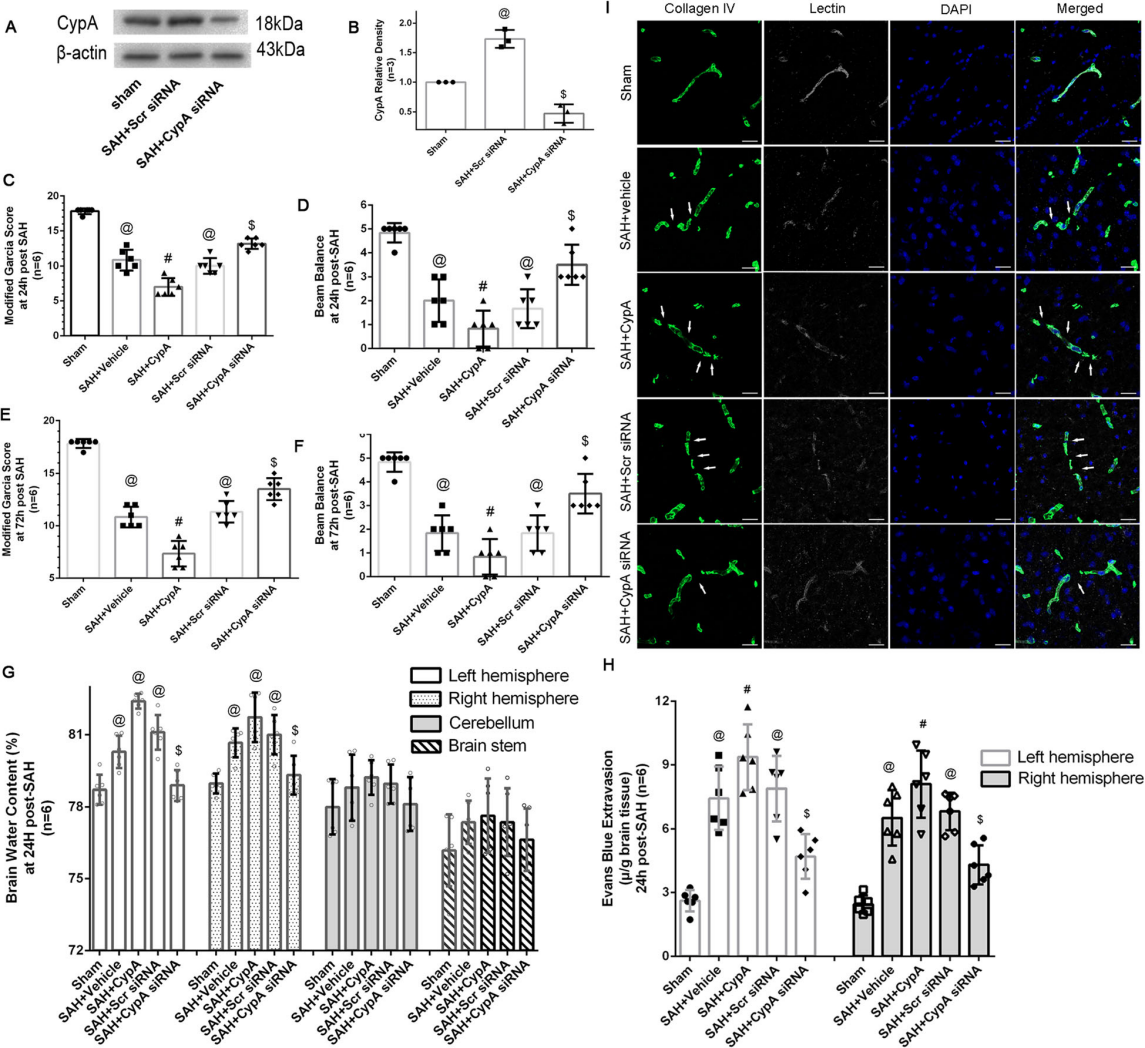
Effects of exogenous CypA and CypA small interfering RNA treatment on blood–brain barrier after subarachnoid hemorrhage. **a** Representative Western blot bands of the inhibition effect of CypA siRNA after SAH. **b** Quantitative analysis of the western blot bands in (**a**). **c** Modified Garcia test and **d** beam balance test results of each group at 24 h after SAH. **e** Modified Garcia test and **f** beam balance test results of each group at 24 h after SAH. **g** Brain water content assessment at 24 h after SAH. **h** Evans blue extravasation evaluation at 24 h after SAH. **i** Representative immunohistochemistry staining slices Collagen IV (Green) and Lectin (White) at 24 h after SAH. Arrow indicates the breakdown of continuous endothelia cell layer. *n* = 6. @: vs. sham *P* < 0.05, #: vs. SAH + vehicle *P* < 0.05, and $: vs. SAH + Scr siRNA *P* < 0.05

The CypA siRNA used in the present study could effectively inhibit the CypA expression after SAH (Fig. 3a, b). And comparisons of the SAH grading score did not reveal significant differences among the groups either at 24 or 72 h after SAH. Compared with the Sham group, all SAH mouse models showed significant neurological impairment as assessed by the modified Garcia test both at 24 and 72 h after SAH induction (Fig. 3c–f). Further analysis indicated that the SAH + CypA mice had more severe neurological deficits at 24 and 72 h after SAH compared with the SAH + vehicle mice (Fig. 3c–f). However, the CypA siRNA treatment showed greater improvements in neurological deficits compared with the SAH + vehicle, SAH + CypA, and SAH + scrambled siRNA groups at both 24 and 72 h after SAH (Fig. 3c–f). For the beam balance test, all SAH mouse models showed significant neurobehavioral dysfunction compared with the Sham group at 24 and 72 h after SAH except the SAH + CypA siRNA group at 72 h after SAH (Fig. 3c–f). Similarly, for the modified Garcia test, mice from the SAH + CypA group exhibited significantly worse neurobehavioral functions compared with the mice from the SAH + vehicle groups (Fig. 3c–f). In addition, the CypA siRNA treatment significantly alleviated neurological impairments at 24 and 72 h after SAH (Fig. 3c–f).

Mice from the SAH + vehicle, SAH + CypA, and SAH + scrambled siRNA groups showed increased brain water content in both hemispheres 24 h after SAH compared with the mice from the Sham group (Fig. 3g). The CypA treatment did not increase the brain water content compared with the SAH + vehicle group, whereas the CypA siRNA pretreatment significantly reduced the brain water content in both hemispheres at 24 h after SAH (Fig. 3g). Furthermore, the SAH mouse models showed more Evans blue extravasation than the mice in the Sham group in both hemispheres (Fig. 3h). The SAH + CypA mice exhibited increased Evans blue leakage compared with the SAH + vehicle mice, whereas the CypA siRNA pretreatment significantly reduced the Evans blue extravasation compared with the SAH + scrambled siRNA group (Fig. 3h). In the immunohistochemical staining images, continuous Lectin-positive endothelial cells and a collagen IV-positive basement membrane were observed in the Sham mice. However, in the SAH + vehicle, SAH + CypA, and SAH + scrambled siRNA groups, the Lectin and collagen IV structures were disrupted at 24 h after SAH, and the CypA siRNA pretreatment effectively reduced these damages (Fig. 3i).

**Fig. 4:**
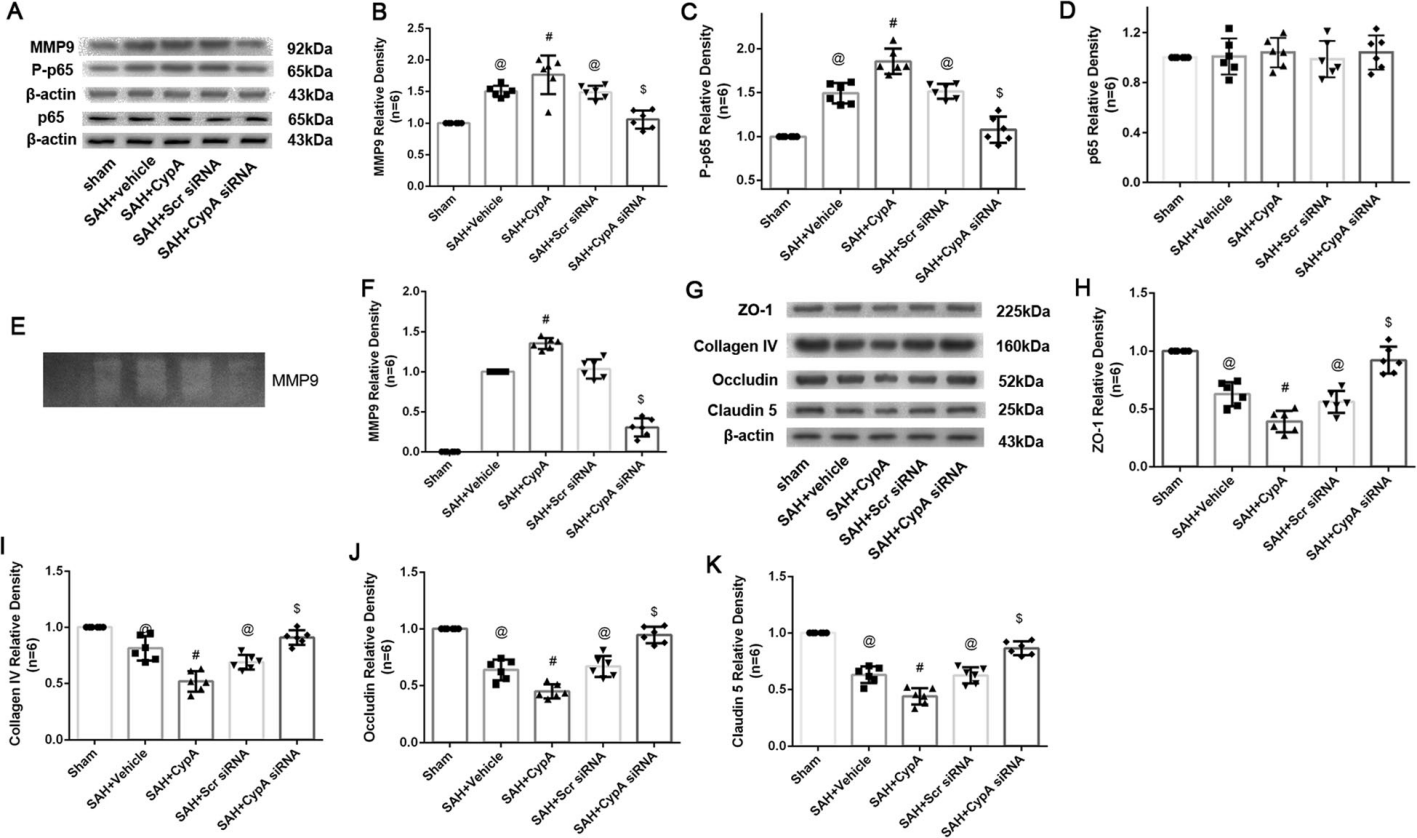
Effects of exogenous CypA and CypA small interfering RNA pretreatment on MMP9 secretion and activities after subarachnoid hemorrhage. **a** Representative western blot bands of MMP9 P-p65and total P=p65from ipsilateral hemisphere after SAH. Quantitative analyses of **b** MMP9, **c** Pp65, and **d** total p-65 expressions from ipsilateral hemisphere after SAH. **e** Representative zymography bands and **f** quantitative analysis of MMP9 activities from ipsilateral hemisphere after SAH. **g** Representative bands and quantitative analysis of (H) ZO-1, **i** Collagen IV, **j** Occludin, and **k** Claudin 5 expressions in ipsilateral hemisphere of brain specimen at 24 h after SAH. Relative densities of each protein have been normalized against the sham group. *n* = 6. @: vs. sham *P* < 0.05, #: vs. SAH + vehicle *P* < 0.05, and $: vs. SAH + scrambled siRNA *P* < 0.05

The expression of MMP9 was significantly increased 24 h after SAH and decreased after the CypA siRNA pretreatment (Fig. 4a, b). Gelatin zymography showed that the MMP9 levels were elevated in the SAH + vehicle, SAH + CypA, and SAH + scrambled siRNA groups compared with the Sham group, and these levels were significantly elevated in the SAH + CypA group compared with the SAH + vehicle and SAH + scrambled siRNA groups (Fig. 4e, f). However, the CypA siRNA pretreatment decreased the MMP9 levels at 24 h after SAH (Fig. 4a, b). Furthermore, the expression of P-p65 was significantly increased 24 h after SAH, whereas the ZO-1, Collagen IV, Occludin, and Claudin 5 levels were significantly reduced at 24 h after SAH (Fig. 4a, c, g–k). While total p65 did not show significant differences among groups. The CypA siRNA pretreatment decreased the expression levels of P-p65 and preserved the ZO-1, Collagen IV, Occludin, and Claudin 5 levels compared with the SAH + vehicle group (Fig. 4a, c, g–k).

### CypA secretion and endocytosis in cultured pericytes

**Fig. 5:**
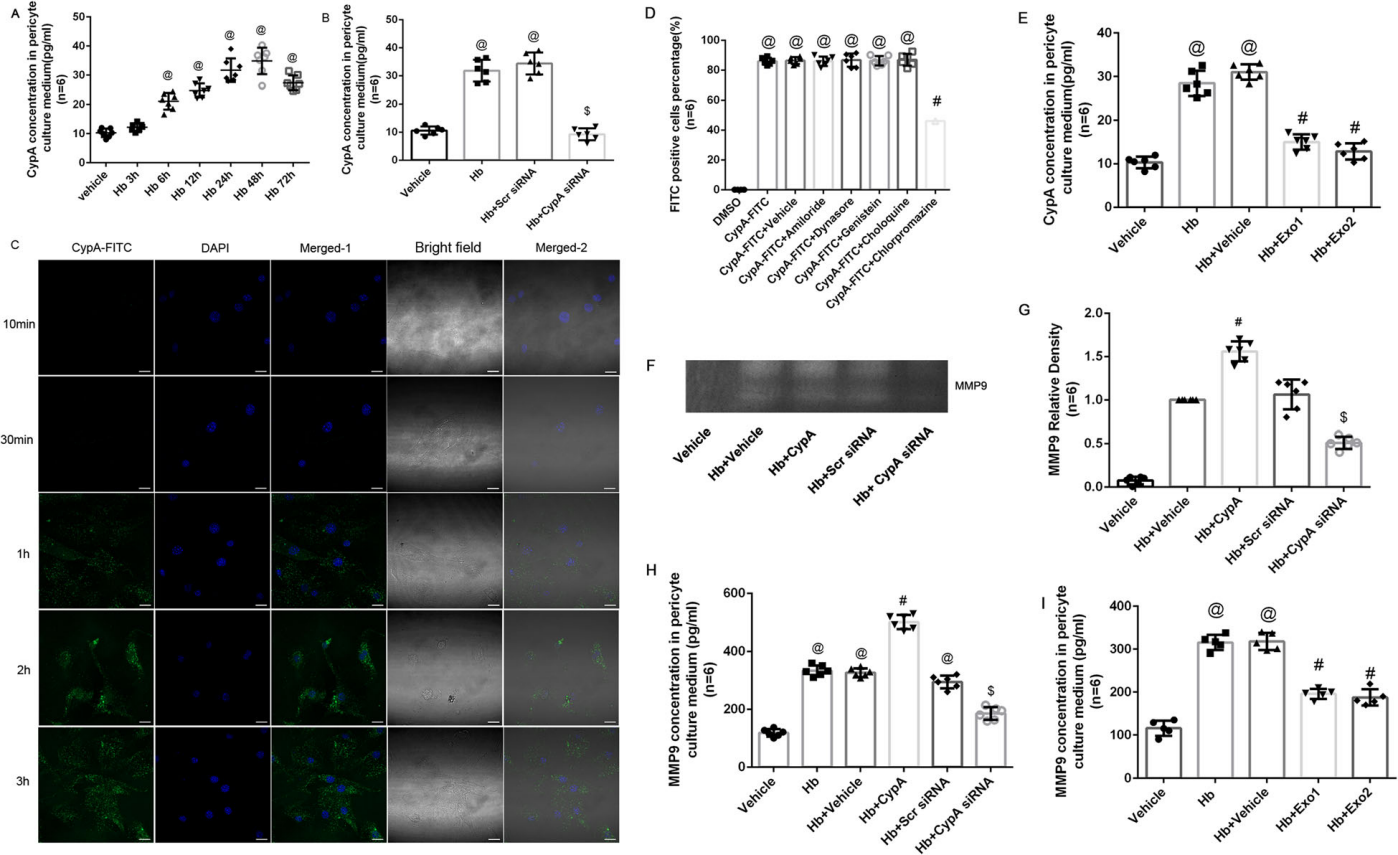
CypA secretion and endocytosis in cultured pericytes. **a** ELISA results of CypA concentrations in pericyte culture media after Hb stimulation. @: vs. sham *P* < 0.05. **b** ELISA results of CypA concentrations in pericyte culture media pretreated with CypA siRNA at 24 h after Hb stimulation. @: vs. vehicle *P* < 0.05, $: vs. Hb + Scr siRNA *P* < 0.05. **c** Representative FITC positive cells in cultured pericyte after exogenous CypA-FITC stimulation. **d** Flow cytometry results of FITC-positive cell percentage after treatment of endocytosis inhibitors and CypA-FITC on cultured pericyte. @: vs. DMSO *P* < 0.05. #: vs. CypA-FITC *P* < 0.05. **e** ELISA results of MMP9 concentrations in pericyte culture media after Hb stimulation. @: vs. vehicle *P* < 0.05, #: vs. Hb + vehicle *P* < 0.05. **f** Representative zymography bands and **g** quantitative analysis of MMP9 activities from ipsilateral hemisphere after SAH. #: vs. Hb *P* < 0.05, $: vs. Hb + Scr siRNA. **h** ELISA results of MMP9 concentrations in pericyte culture media after Hb stimulation. @: vs. vehicle *P* < 0.05, #: vs. Hb + vehicle *P* < 0.05, $: vs. Hb + Scr siRNA *P* < 0.05. **i** ELISA results of MMP9 concentrations in pericyte culture media after Hb stimulation. @: vs. vehicle *P* < 0.05, #: vs. Hb + vehicle *P* < 0.05. *n* = 6

To determine the role of CypA in the pericyte- and MMP9-associated blood-–brain barrier disruption, we cultured primary pericytes and evaluated the intracellular and extracellular CypA expression. Intracellular CypA expression in the cultured pericytes was significantly increased at 6 to 72 h after Hb stimulation, with the peak expression occurring at 24 to 72 h after stimulation (Fig. 1c), which was confirmed by the immunohistochemical staining of CypA in the cultured pericytes at 24 h after Hb stimulation (Fig. 1d). In addition, ELISA data indicated secreted CypA in the culture media was significantly increased at 6 to 72 h after Hb stimulation, and peak at 24 to 48 h (Fig. 5a). The CypA concentrations in the culture media were reduced at 24 h after Hb stimulation in the CypA siRNA-pretreated group compared with the scrambled siRNA-pretreated group (Fig. 5b).

In the present study, exogenous CypA was conjugated with FITC to investigate the endocytosis of CypA in the cultured pericytes. Confocal microscopy observations indicated that CypA-FITC was endocytosed into the cultured pericytes 1 h after CypA-FITC incubation and continued for up to 3 h (Fig. 5c), indicating that cypa could be endocytosed by pericyte physiologically. Further flow cytometry analyses indicated that this endocytosis could be inhibited by chlorpromazine but not by amiloride, dynasore, genistein, or chloroquine (Fig. 5d).

Secretion inhibitor Exo1 and Exo2 was used to study process of CypA secretion from pericyte. We found both Exo1 and Exo2 reducing CypA in medium after Hb stimulation (Fig. 5e).

Furthermore, the MMP9 concentrations and activities in culture media were significantly increased after Hb simulation versus vehicle group, and even much higher in Hb + CypA group versus solo Hb stimulation group. By pretreated with CypA siRNA, the MMP9 concentrations and activities in culture media were significantly lower than Hb group and Hb + CypA group (Fig. 5f–h). Both Exo1 and Exo2 treatment significantly lowered MMP9 concentration in cultured medium post Hb stimulation (Fig. 5i).

### CD147 expressions in pericytes after SAH

**Fig. 6:**
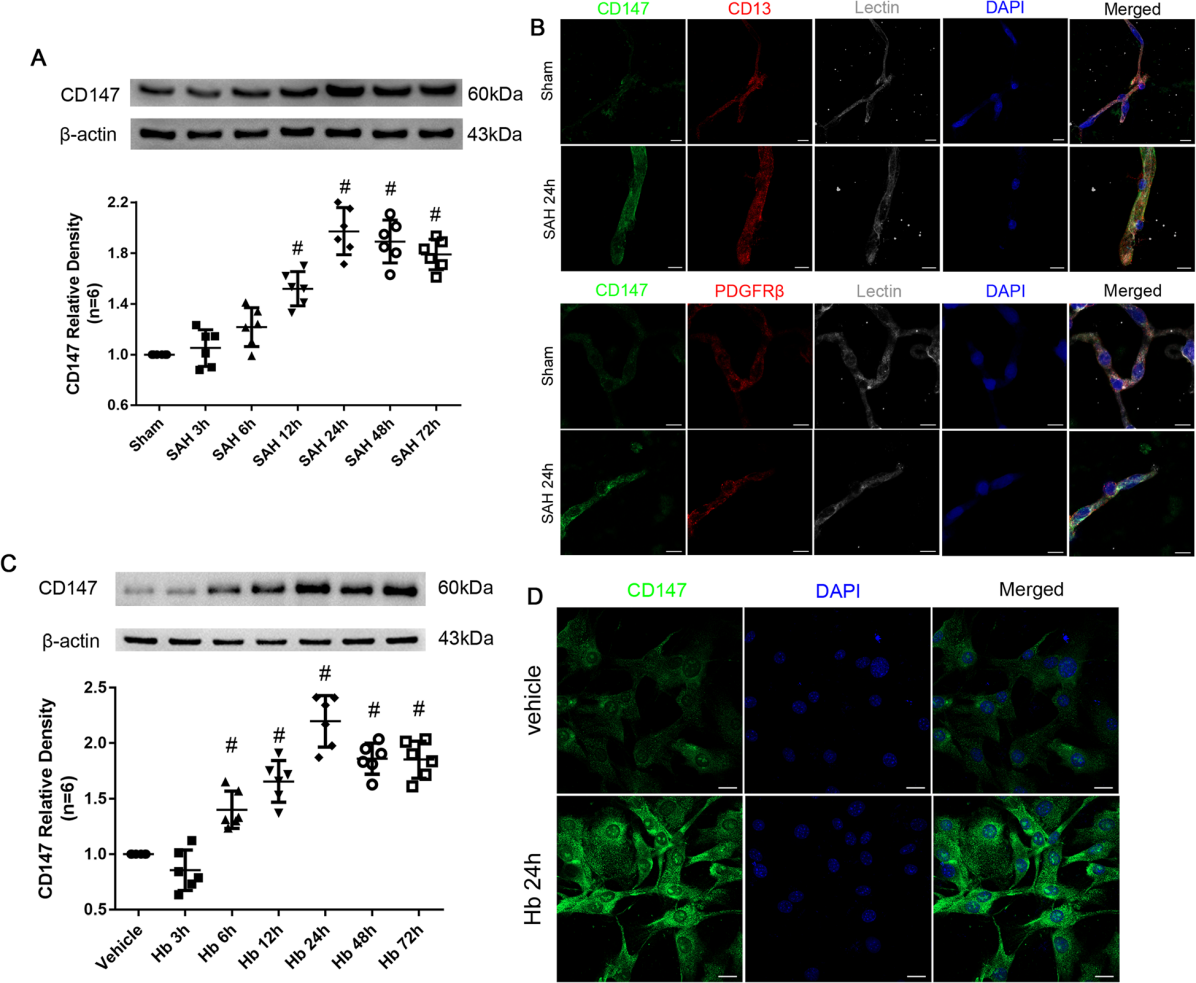
Time course of endogenous CD147 in pericyte after subarachnoid hemorrhage. **a** Representative western blot bands and quantitative analysis of CD147 from ipsilateral hemisphere after SAH. **b** Representative immunohistochemistry staining slices of CD147 (Green), PDGFRβ/CD13 (Red), and Lectin (White) at 24 h after SAH. **c** Representative western blot bands and quantitative analysis of CD147 expressions in cultured pericytes after Hb stimulation. **d** Representative immunohistochemistry staining of CD147 (Green) in cultured pericytes at 24 h after Hb stimulation

CD147 has been reported to serve as the receptor for CypA; therefore, we also evaluated CD147 expression in the pericytes after SAH induction. We found that CD147 was significantly increased in cerebral microvessels after SAH except at 6 h, and a peak was observed at 24 h, and it was sustained until 72 h after SAH as confirmed by immunohistochemical staining of Lectin and PDGFRβ/CD13 at 24 h after SAH (Fig. 6a, b). Similarly, the cultured pericytes also overexpressed CD147 from 12 to 72 h after Hb stimulation, with a peak observed at 24 h, and the expression pattern was also confirmed by immunohistochemical staining with a CD147 antibody (Fig. 6c, d).

### CD147 inhibition reduced MMP9 secretion without affecting CypA endocytosis

**Fig. 7:**
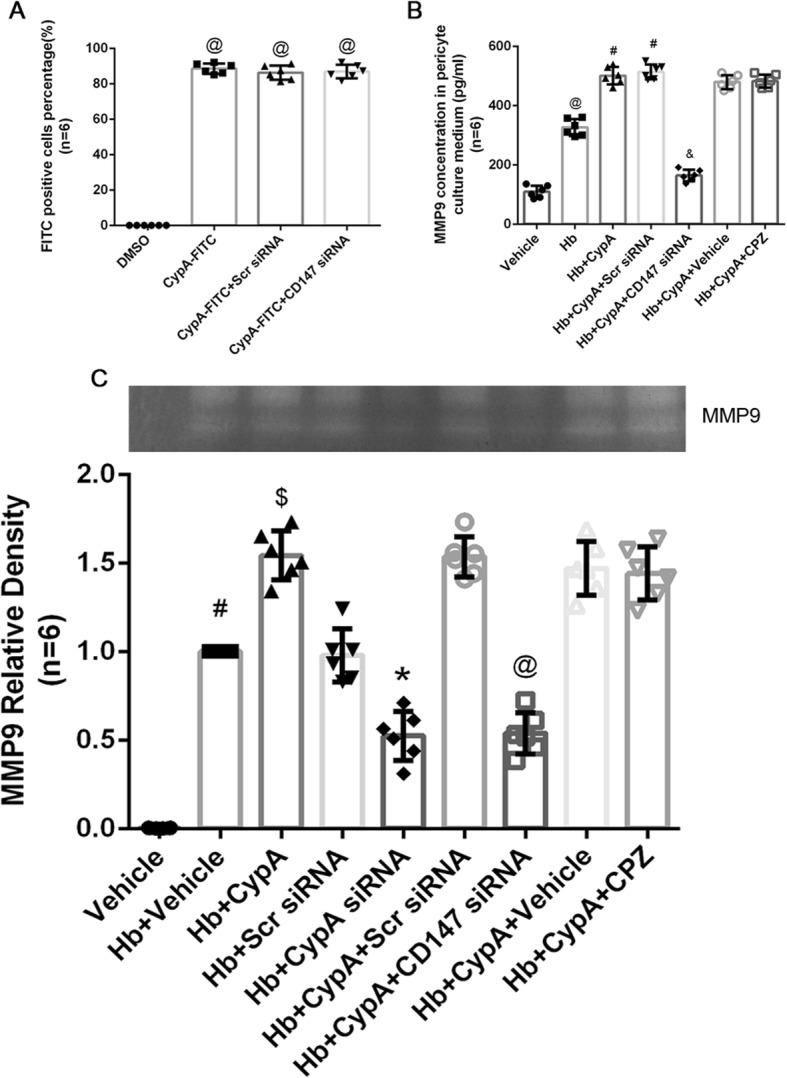
CD147 inhibition reduced MMP9 secretion without effecting CypA endocytosis. **a** Representative FITC-positive cell percentage after treatment of CD147 siRNA and CypA-FITC on cultured pericyte. *n* = 6. @: vs. DMSO *P* < 0.05. **b** ELISA results of MMP9 concentrations in pericyte culture media after exogenous CypA, CD147 siRNA, and chlorpromazine (CPZ) treatment. *n* = 6. @: vs. Hb *P* < 0.05. #: vs. Hb + CypA *P* < 0.05, &: vs. Hb + CypA + Scr siRNA *P* < 0.05. $: vs. Hb + CypA + vehicle *P* < 0.05. **c** Representative zymography bands and quantitative analysis of MMP9 of cultured pericyte supernatant after exogenous CypA, CD147 siRNA, and CPZ treatment. Relative densities of each group have been normalized against the first group. *n* = 6. #: vs. Hb + vehicle *P* < 0.05. $: vs. Hb + CypA *P* < 0.05, *: vs. Hb + Scr siRNA *P* < 0.05, @: vs. Hb + CypA siRNA *P* < 0.05

Significant differences in the FITC-positive cells percentage of CypA-FITC were not observed in the CD147 siRNA-pretreated cultured pericytes at 1 h after incubation (Fig. 7a). However, the MMP9 concentration in the culture media was significantly increased at 24 h after Hb + CypA incubation and reduced by the CD147 siRNA pretreatment (Fig. 7b). In addition, chlorpromazine had an inhibitory effect on CypA endocytosis, but it did not reduce MMP9 concentrations in the supernatant of the culture media compared with the Hb + CypA + vehicle group (Fig. 7b). Gelatin zymography of the media supernatants also showed that Hb + CypA incubation increased the pro-MMP9 activity compared with the Hb + vehicle group, whereas the Hb + CypA siRNA group showed less pro-MMP9 activity than the Hb + scrambled siRNA group. CD147 siRNA significantly reduced the pro-MMP9 activity following Hb + CypA incubation compared with the Hb + CypA + CD147 siRNA group, whereas significant differences were not observed between the Hb + CypA + vehicle and Hb + CypA + chlorpromazine groups (Fig. 7c, d).

### Effects of exogenous recombinant CypA and specific inhibition of CD147 expression after SAH

**Fig. 8:**
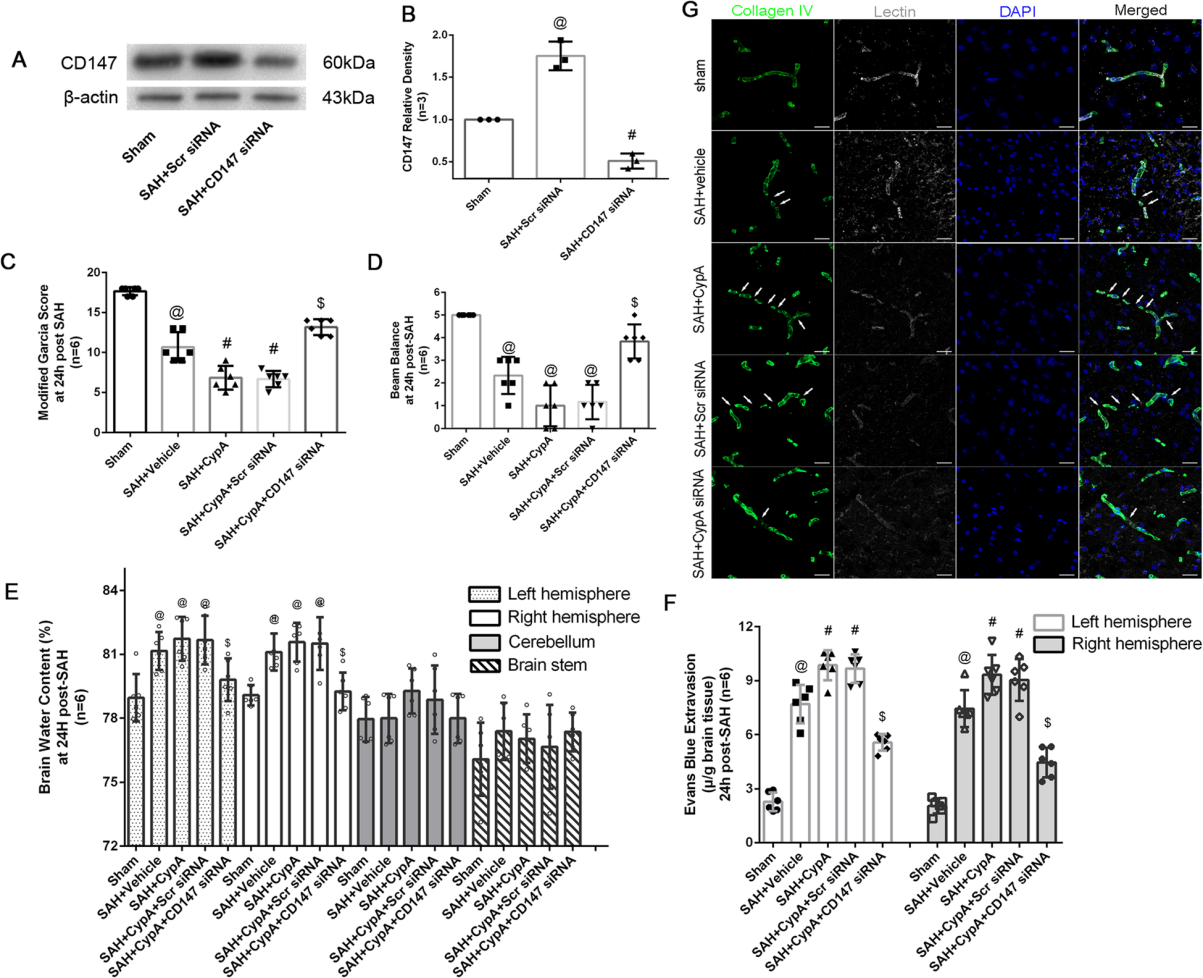
Effects of exogenous CypA and CD147 small interfering RNA treatment on blood–brain barrier after subarachnoid hemorrhage. **a**, **b** Representative Western blot bands and quantitative analysis of the inhibition effect of CD147 siRNA after SAH. **c** Modified Garcia test and **d** beam balance test results of each group at 24 h after SAH. **e** Brain water content assessment at 24 h after SAH. **f** Evans blue extravasation evaluation at 24 h after SAH. **g** Representative immunohistochemistry staining slices Collagen IV (Green) and Lectin (White) at 24 h after SAH. @: vs. sham *P* < 0.05, #: vs. SAH + vehicle *P* < 0.05 and $: vs. SAH + CypA + Scr siRNA *P* < 0.05

The CD147 siRNA used in the present study could effectively inhibit the CD147 expression after SAH (Fig. 8a, b), and comparisons of the SAH grading score did not reveal significant differences among the groups at 24 h after SAH. Mice from the SAH + CypA + CD147 scrambled siRNA and SAH + CypA + CD147 siRNA groups showed significant neurological impairment during the modified Garcia test and the beam balance test at 24 h after SAH compared with the Sham group (Fig. 8c, d). However, the SAH + CypA + CD147 siRNA group showed alleviated neurological impairment compared with the SAH + CypA + CD147 scrambled siRNA groups (Fig. 8c, d). In addition, the SAH + CypA + CD147 scrambled siRNA group showed increased brain water content in both hemispheres at 24 h after SAH (Fig. 8e). The SAH + CypA + CD147 siRNA pretreatment significantly reduced the brain water content in both hemispheres at 24 h after SAH (Fig. 8e). Furthermore, the SAH + CypA + CD147 siRNA group showed less Evans blue extravasation than the SAH + CypA + CD147 scrambled siRNA group in both hemispheres at 24 h after SAH, although the Evans blue extravasation level was still higher than that in the Sham group (Fig. 8f).

**Fig. 9:**
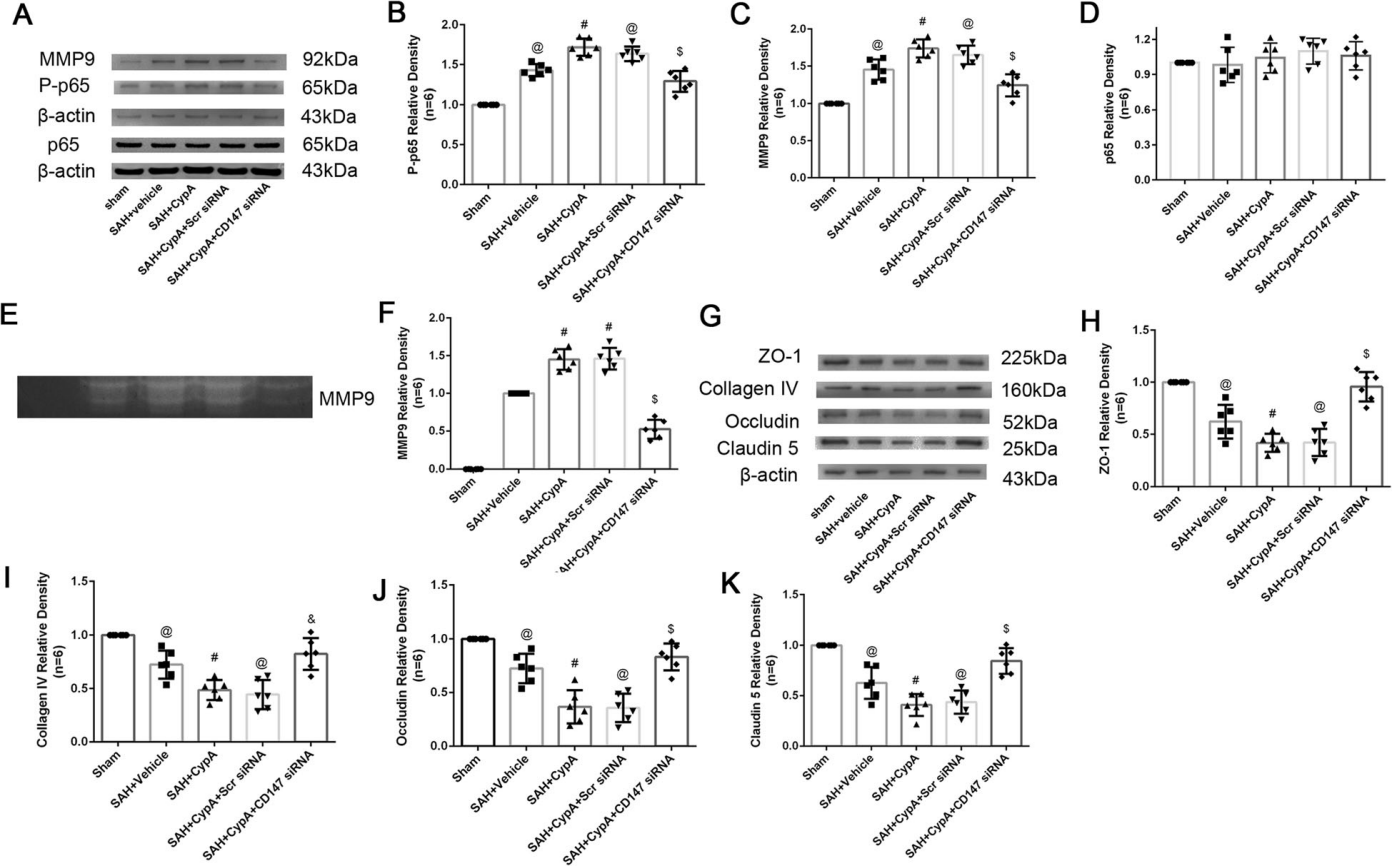
Changes of P-p65, MMP9, Collagen IV, and tight junction proteins expression after treatment at 24 h post subarachnoid hemorrhage. **a** Representative Western blot bands of MMP9 P-p65 and total p65 from ipsilateral hemisphere after SAH. Quantitative analyses of **b** MMP9, **c** P-p65, and total p65 **d** expressions from ipsilateral hemisphere after SAH. **e** Representative zymography bands and **f** quantitative analysis of MMP9 activities from ipsilateral hemisphere after SAH. **g** Representative bands and quantitative analysis of **h** ZO-1, **i** Collagen IV, **j** Occludin, and **k** Claudin 5 expressions in ipsilateral hemisphere of brain specimen at 24 h after SAH. Relative densities of each protein have been normalized against the sham group. *n* = 6 @: vs. sham *P* < 0.05, #: vs. SAH + Vehicle *P* < 0.05, and $: vs. SAH + CypA + Scr siRNA *P* < 0.05

Immunohistochemical staining showed that the continuous endothelial cell layer (Lectin) and basement membrane (collagen IV) were disrupted in the SAH + CypA + CD147 siRNA scramble group at 24 h after SAH, which was similar to the phenomenon observed in the SAH + vehicle and SAH + CypA groups; however, the CD147 siRNA pretreatment effectively reduced those damages (Fig. 8g). Further analysis indicated that the expression of P-p65 and MMP9 was significantly increased and total p65 was not altered at 24 h after SAH (Fig. 9a–d). In addition, gelatin zymography confirmed that the tight junctions and basement membranes were disrupted by MMP9 activity after SAH but alleviated by the CD147 siRNA pretreatment compared with the SAH + CypA + CD147 scrambled siRNA group (Fig. 9e, f). And the ZO-1, collagen IV, Occludin, and Claudin 5 was significantly reduced at 24 h after SAH (Fig. 9g–k). The CD147 siRNA pretreatment decreased the expression levels of P-p65 and MMP9 and preserved the expression levels of ZO-1, Collagen IV, Occludin, and Claudin 5 compared with that of the SAH + CypA + CD147 siRNA group (Fig. 9g–k).

## Discussion

**Fig. 10:**
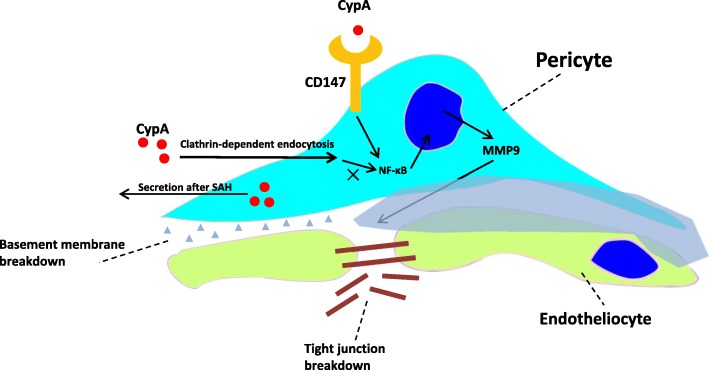
CypA signal in pericytes induce rapid BBB disruption via CD147/NF-κB/MMP9 pathway after SAH

The present study demonstrated that both intracellular CypA and CypA secretion increased after SAH and could activate its receptor CD147 and the downstream NF-κB inflammatory pathway to induce MMP9 expression and proteolytic functions for the degradation of endothelium tight junction proteins and basal membranes. CypA served as an autocrine or a paracrine ligand for its receptor, CD147 (Fig. 10). Although CypA could be endocytosed by pericytes, the specific endocytosis inhibitor chlorpromazine did not have an effect on MMP9 activation following blood–brain barrier disruption. However, the specific knockdown of CD147 could reverse the harmful effects of CypA expression in the pericytes on the blood–brain barrier integrity after SAH.

The blood–brain barrier is a highly selective neurovascular structure that can prevent harmful substances from entering the brain [19]. After SAH and other acute central nervous system injuries, increased blood–brain barrier permeability could lead to worse secondary pathophysiological changes by exposing neurons and other support cells in the vascular neural network to blood metabolites, and it could also lead to vasogenic brain edema, which causes further deterioration [20, 21]. Based on the intimate connections between pericytes and endothelial cells, the pericytes have been shown to play a substantial role in the development of blood-brain barrier tight junctions and paracellular permeability [22]. Pericytes can form intercellular blood–brain barrier tight junctions [23]. In addition, pericytes also contribute to basal lamina formation by synthesizing collagen type IV, glycosaminoglycans, and laminin [24]. An inability to recruit pericytes to the endothelium in PDGF-B knockout mice results in severe vascular abnormalities, plasma leakage, and microaneurysm formation, indicating that pericytes maintain blood–brain barrier functions [3, 25]. However, the response of normally developed pericytes in the adult brain to the pathophysiological changes after SAH is not well understood. Because of the well-known pearl-like contraction in SAH patients with vasospasm, previous researchers as well as our group have studied the contractile functions of pericytes and their role in regulating microcirculation after SAH [6]. In the present study, our data demonstrated the harmful effects of pericytes on the blood–brain barrier integrity after SAH. Cultured pericytes have recently been shown to produce matrix metalloproteinases after thrombin stimulation, and matrix metalloproteinases are key mediators of blood–brain barrier disruptions after SAH [26]. In addition, a recent study suggested that pericytes may be harmful for neurovascular integrity, which results in neuronal degeneration in apolipoprotein E-deficient mice [7]. Additional direct evidence was obtained with a two-photon microscope, and the results indicated that pericytes contribute to rapid and localized proteolytic degradation around pericyte somata during ischemia [8].

Cyclophilins are a protein family of highly conserved and ubiquitous proteins with peptidylprolyl isomerase activity [27]. The most distributed cyclophilin in humans is CypA, which is primarily expressed intracellularly and could be secreted from cells in response to various inflammatory stimulations after hypoxia, infection, and oxidative stress [27, 28]. Previous studies have demonstrated that CypA activity is a modulator of CD4^+^ T cell signal transduction following cytokine production [29]. However, the exact mechanism underlying CypA secretion from the intracellular to extracellular space has not been well described. Previous studies have shown that CypA can be secreted from vascular smooth muscle cells via Rho guanine triphosphatease-myosin II activation/actin remodeling-regulated vesicle transport [30, 31]. Another study has shown that the acetylation of CypA is required for its secretion into the extracellular space in vascular smooth muscle cells in response to oxidative stress [32]. And neuron was also found to express CypA [33]. Because reactive oxygen species could be generated in the central nervous system after SAH [34], we hypothesized that CypA may be secreted from pericytes and other cells in response to oxidative stress post-SAH. Exo 1-inhibited vesicular traffic from the endoplasmic reticulum to the Golgi might by interfere with GEF-stimulated GDP/GTP exchange on ARF [35]; Exo2 disrupts the Golgi apparatus and interferes the secretory cargo exit from the endoplasmic reticulum [36]. Our data showed both Exo1 and Exo2 could efficiently reduce CypA secretion from pericyte, but whether these effects were identical to reported mechanism is unknown; further study is needed to illustrate to detail mechanism.

Our results indicated that extracellular CypA can be endocytosed by pericytes, which is a finding that has rarely been reported. M Carpentier et al. once illustrated that cyclophilin B could bind to two types of binding sites at the surface of capillary endothelial cells and showed that one type is involved in the endocytosis process [37]. Sanglifehrin A, a CypA-binding immunosuppressive drug, could reduce Lectin-mediated endocytosis in dendritic cells, which may occur via the decreased expression of C-type Lectins [38, 39]. A subsequent study demonstrated that the endocytosis gene END3 plays a pivotal role in the internalization of extracellular proteins/vesicles in response to glucose, which could rapidly decrease the extracellular CypA level under proper glucose conditions, which presumably occurs by enhancing CypA endocytosis [40]. However, additional evidence on the regulation of CypA endocytosis is required. The present study sought to investigate the role of CypA signaling in pericyte-associated blood–brain barrier disruption after SAH, and our data demonstrated that chlorpromazine (chlorpromazine: an inhibitor for clathrin-dependent endocytosis, but not by amiloride: macropiocytosis, dynasore: an inhibitor for dynamin-dependent endocytosis, genistein: an inhibitor for caveolin-dependent endocytosis, or chloroquine:an inhibitor for low pH-dependent endocytosis) could effectively inhibit CypA endocytosis by pericytes and showed that this process was not associated with the MMP9-mediated blood–brain barrier disruption after SAH. We focus on whether extracellular CypA induce MMP9 expression but not which specific progress of endocytosis CypA enter into pericyte.

However, the well-known receptor CD147, which is also known as the extracellular matrix metalloproteinase inducer (EMMPRIN), mediated this pathophysiological process [41]. CD147 is expressed in various types of cells and can crosstalk with integrin, annexin, caveolin, osteopontin, cyclophilin, etc. [41–45]. However, the functions of CD147 require further clarification. Previous studies have indicated that CD147 is expressed on the surface of normal endothelial cells in the central nervous system and showed that its expression could be induced in Alzheimer’s disease, in brain tumors, and following ischemic brain injury [41, 42, 45, 46]. Because CD147 was demonstrated to induce MMP-1 expression in co-cultured tumor cells and fibroblast cells, it was considered an MMP inducer [41, 45]. Further analysis showed that CD147 colocalized with MMP9 protein and displayed activity in experimental autoimmune encephalomyelitis brain tissues. After treatment with CD147-blocking antibodies, the improved outcome in the experimental autoimmune encephalomyelitis mice was associated with diminished MMP9 proteolytic activity [42]. More recently, CypA-CD147 signaling was demonstrated to be neuroprotective in both in vitro oxidative and ischemic neuron injury as well as in vivo early brain injury after SAH [33, 47]. S Chen et al. showed that MMPs are also involved in CypA/CD147-induced blood–brain barrier breakdown after SAH [48]. Our experiments provide direct evidence to show that CypA induced pericyte-associated blood–brain barrier disruption after SAH via the NF-κB/MMP9 pathway.

Nevertheless, this study has limitations. The focus was on the CypA-CD147 pathway; thus, NF-κB translocation was not studied in detail. NF-κB translocation, which elevates MMP9 transcription, has been well established in other studies. In addition, CypA may be located in the cytoplasm and the extracellular region and could affect surrounding cells. Consistent with previous studies, we found that pericytes release CypA as an autocrine signaling molecule. However, whether other types of cells secrete CypA and have effects on pericytes after SAH are unknown. Other unknown mechanisms of CypA cannot be ruled out; thus, in our future studies, the expression of CypA in other cell types and the mechanisms of CypA will be clarified.

## Conclusions

This study demonstrated for the first time that CypA is elevated in the pericytes of the brain after SAH, and extraneous recombinant CypA damages the blood–brain barrier and aggravates the neurological outcome. This process was potentially mediated by CD147, which may promote NF-κB nuclear translocation, MMP9 secretion, and junction protein degradation in the brain. By targeting CypA and pericytes, our present data and further translational studies may provide new insights on the management of SAH patients.

## Supplementary information

**Additional file 1: Figure S1.** The experimental design of present study.

## Data Availability

Data sharing is not applicable to this article as no datasets were generated or analyzed during the current study.

## Abbreviations

ANOVA: Analysis of variance
CPZ: Chlorpromazine
CypA: Cyclophilin A
EBI: Early brain injury
FBS: Fetal bovine serum
Hb: Oxyhemoglobin
KO: Knockout
PBS: Phosphate-buffered saline
SAH: Subarachnoid hemorrhage
Scr siRNA: Scrambled small interference RNA
siRNA: Small interference RNA
TJs: Tight junctions
