# Supplementary figures and images for: Cyclophilin a signaling induces pericyte-associated blood-brain barrier disruption after subarachnoid hemorrhage
Source: J Neuroinflammation. 2020 Jan 11;17:16. doi: 10.1186/s12974-020-1699-6 (PMC6954572; doi:10.1186/s12974-020-1699-6)

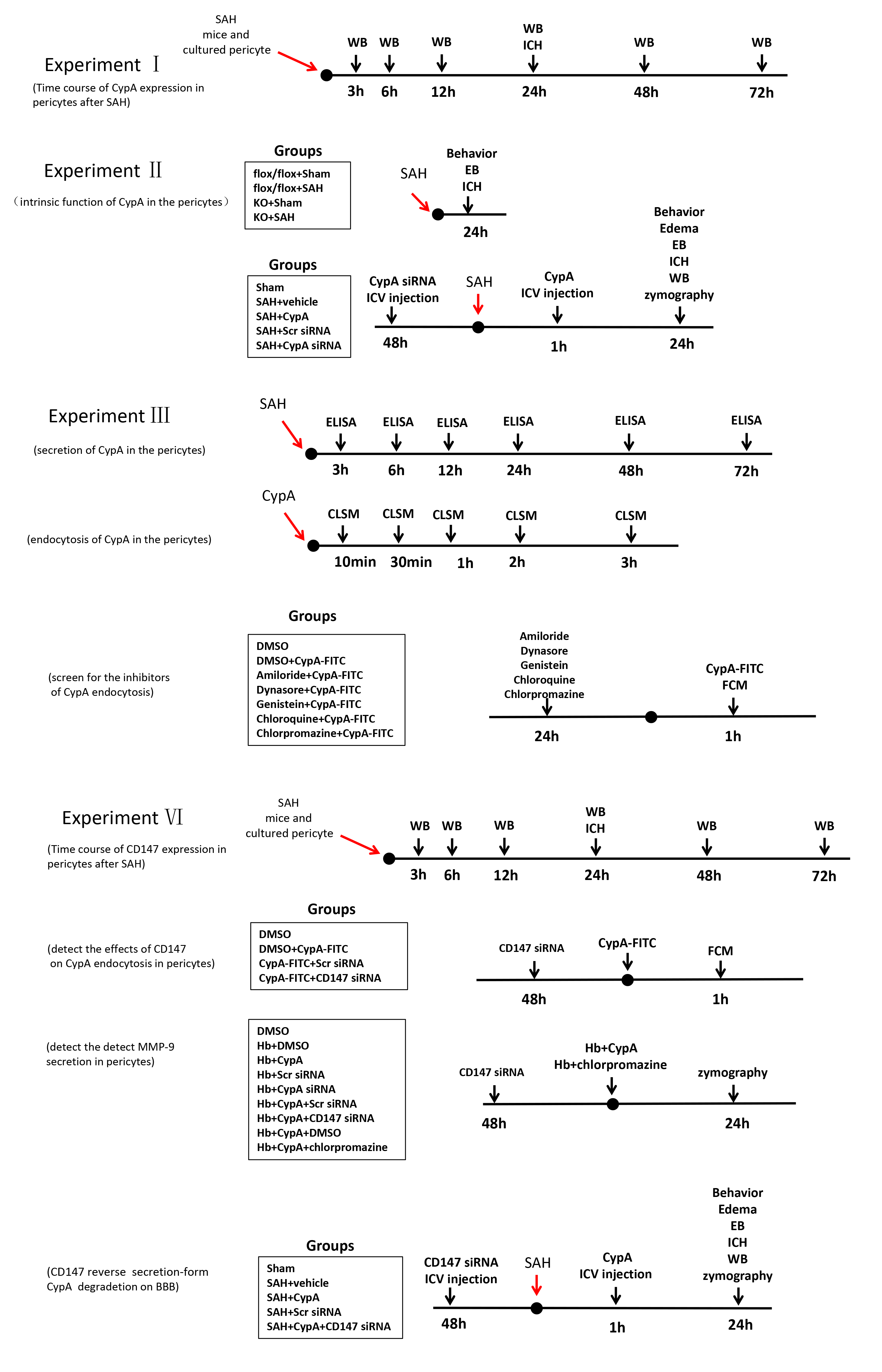

Supplement: Supplementary file 1 — Additional file 1: Figure S1. The experimental design of present study. [file 12974_2020_1699_MOESM1_ESM.tif]
